# A Rapid and Improved Method to Generate Recombinant Dengue Virus Vaccine Candidates

**DOI:** 10.1371/journal.pone.0152209

**Published:** 2016-03-23

**Authors:** Dhanasekaran Govindarajan, Liming Guan, Steven Meschino, Arthur Fridman, Ansu Bagchi, Irene Pak, Jan ter Meulen, Danilo R. Casimiro, Andrew J. Bett

**Affiliations:** 1 Merck Research Laboratories, Merck & Co., Kenilworth, NJ, United States of America; 2 Immune Design Corporation, Seattle, WA, United States of America; University of Texas Medical Branch, UNITED STATES

## Abstract

Dengue is one of the most important mosquito-borne infections accounting for severe morbidity and mortality worldwide. Recently, the tetravalent chimeric live attenuated Dengue vaccine Dengvaxia^®^ was approved for use in several dengue endemic countries. In general, live attenuated vaccines (LAV) are very efficacious and offer long-lasting immunity against virus-induced disease. Rationally designed LAVs can be generated through reverse genetics technology, a method of generating infectious recombinant viruses from full length cDNA contained in bacterial plasmids. *In vitro* transcribed (IVT) viral RNA from these infectious clones is transfected into susceptible cells to generate recombinant virus. However, the generation of full-length dengue virus cDNA clones can be difficult due to the genetic instability of viral sequences in bacterial plasmids. To circumvent the need for a single plasmid containing a full length cDNA, *in vitro* ligation of two or three cDNA fragments contained in separate plasmids can be used to generate a full-length dengue viral cDNA template. However, *in vitro* ligation of multiple fragments often yields low quality template for IVT reactions, resulting in inconsistent low yield RNA. These technical difficulties make recombinant virus recovery less efficient. In this study, we describe a simple, rapid and efficient method of using LONG-PCR to recover recombinant chimeric Yellow fever dengue (CYD) viruses as potential dengue vaccine candidates. Using this method, we were able to efficiently generate several viable recombinant viruses without introducing any artificial mutations into the viral genomes. We believe that the techniques reported here will enable rapid and efficient recovery of recombinant flaviviruses for evaluation as vaccine candidates and, be applicable to the recovery of other RNA viruses.

## Introduction

Dengue viruses (DENV) are mosquito-borne viruses belonging to the family *Flaviviridae* which includes several other medically-important viruses such as Yellow fever virus (YFV), Japanese encephalitis virus (JEV), Tick-borne encephalitis virus (TBEV), West Nile virus (WNV) and Zika virus (ZV). DENV are enveloped viruses that contain non-segmented positive-sense RNA genomes of ~11kb in length. There are four serotypes of DENV (DENV-1, DENV-2, DENV-3 and DENV-4) that cause diseases ranging from mild-flu like illness to more severe manifestations such as hemorrhagic fever and/or Dengue shock syndrome. It has been estimated that 4 billion people around the globe are at risk of infection with dengue and that approximately 390 million infections occur worldwide annually, of which 96 million are symptomatic cases [1, 2]. Though licensed prophylactic vaccines are available for YFV, JEV and TBEV, there was no licensed vaccine available for dengue until Dengvaxia^®^ was approved in several dengue endemic countries in December 2015 [3]. Dengvaxia^®^ uses the yellow fever virus as a backbone to carry the prM and E genes of dengue viruses 1–4 (CYD-TDV). Though clinical trials of the CYD-TDV vaccine demonstrated protection against severe dengue, the overall vaccine efficacy was limited by DENV serotype, serostatus at vaccination, region and age of vaccinees [4]. For this reason several other dengue vaccine candidates are still in development [5–7]. Currently no vaccine is available to protect against Zika virus infection.

Live attenuated vaccines (LAV) are very efficacious and offer long-lasting immunity against viral diseases including major flavivirus-induced diseases. The Yellow-fever 17D LAV is one of the most successful flavivirus vaccines and has proven to be safe and offers long-lasting immunity. It has been shown that YFV-17D LAV-induced immunity can provide protection for at least 10 years, and up to 45 years in some populations [8]. Several hundred million doses of the YF-17D vaccine have been administered over the past 75 years. Similarly, the LAV (strain SA-14-14-2) for JEV is efficacious and has been used extensively in China [8]. Historically, LAVs have been produced through empirical means. However, with the advent of recombinant DNA technology and infectious clone systems, it is now possible to generate recombinant flaviviruses entirely from cloned cDNA using reverse genetics. Reverse genetics systems offer an excellent means to generate LAVs through rational design. Among flaviviruses, the first reverse genetics system was established for YFV [9]. Though the authors were unsuccessful at constructing a stable full-length infectious clone for YFV due to stability problems in the bacterial host, they recovered recombinant YFV by employing a two-plasmid system. This system, though functional, is technically challenging and involves the in-vitro ligation of two plasmid fragments encompassing the entire genome of YFV-17D, which is then used to generate RNA transcripts in vitro. In some cases, full-length clone generation was achieved by assembling flavivirus genomes in yeast and then propagating in *E*. *coli* using shuttle vectors and homologous recombination [10–12]. Additionally, the use of low copy number plasmids and specialized bacterial strains have offered some improvement in generating YF-17D infectious clones with improved stability [13].

Recombinant chimeric YF-17D based vaccine vectors in which the prM and E genes of a heterologous flavivirus are expressed in the YF-17D backbone have been used to generate LAVs for JEV, WNV, DENV and Modoc virus [14–17]. This was the strategy employed in the development of the four YF-17D-Dengue (CYD) viruses in Dengvaxia® [15, 18]. The generation of the chimeric YF-17D-Dengue (CYD) viruses proved difficult due to severe stability issues in *E*. *coli*. Cumbersome steps such as the ligation of two or even three DNA fragments were required to successfully recover the recombinant viruses [15]. Such technical difficulties pose a serious disadvantage in studying the molecular biology of YF-17D or other flaviviruses and, also for the development of flavivirus LAV vaccine candidates. In this study, we describe a simple, rapid and efficient method using LONG-PCR to generate recombinant CYD viruses that could be used as potential dengue vaccine candidates. Four of the eight designed recombinant CYD viruses were successfully recovered using previously known methods while the remaining four could only be recovered using the LONG-PCR method. Using this method, we were able to efficiently generate viable recombinant viruses without introducing any artificial mutations into the viral genomes. Furthermore, when rhesus macaques were vaccinated with tetravalent formulations of these recombinant viruses they generated strong neutralizing antibody responses against each dengue serotype. We believe that the techniques reported here will enable rapid and efficient recovery of recombinant flaviviruses for virological evaluation or as vaccine candidates.

## Materials and Methods

### Animal ethics statement

All animal studies were performed at the New Iberia Research Center (New Iberia, LA). All experimental procedures, reviewed and approved by the Institutional Animal Care and Use Committee of Merck and Co. Inc (approval no. 2016-600680-Jul) and New Iberia Primate Research Center (approval no.2015-8741-055), were conducted by licensed veterinarians and research staff. Standard cage sizes were 4.3 sq ft. for animals up to 10kg, unless the animal

was taller or longer and would benefit from a larger cage, and 6.0 sq ft. for animals up to 15kg. Socially housed animals were placed in side by side enrichment cages of appropriate size, in climate-controlled rooms (70–82°F) with a 12 h light/dark cycle. The monkeys were fed daily, ad libitum, with specialized monkey chow and fruits. Environmental enrichment was provided to the study animals as part of the New Iberia Research Center's Plan for Environmental Enhancement and Behavioral Management. The standard cage enrichment for indoor-housed animals included maniuplanda (a freely moving toy), a hanging device, and a perch. Animals were monitored daily for clinical signs of disease/discomfort, adverse effect and/or variances from species-specific normal behavior during the entire course of the study. The animals were sedated with Ketamine (5–30 mg/kg, IM) for vaccination, and blood collection. At the completion of the study, all the animals were released back into the colony and no animal was sacrificed during or at the end of the study.

### Cells and viruses

Vero cells (ATCC CCL-81) were maintained in Medium 199 (Life Technologies) supplemented with 10% heat-inactivated fetal bovine serum (HyClone), glutamine (Mediatech) and penicillin-streptomycin (Mediatech). Virus amplification, virus titration and virus neutralization assays were performed on Vero cells. Viruses used in the assays described here were DENV1 (strain WestPac-74), DENV2 (strain S18603), DENV3 (strain CH53489), and DENV4 (strain TVP-360). These viruses were kindly provided by Alan Barrett (University of Texas Medical Branch, Galveston, TX) and belong to a panel of viruses widely used by several reference laboratories and vaccine developers [19]. The viruses were isolated from human subjects and a detailed history of their origin is described elsewhere [20]. Viruses were individually amplified in Vero cells cultured at 37^0^ C at low multiplicities (0.01) of infection. Supernatant from infected cultures was harvested 5–6 days post-infection, clarified at 1000xg for 10 min, divided into 0.2–0.5 ml aliquots, flash-frozen on dry ice, and stored at -70^0^ C for future use.

### Yellow-fever dengue chimeric constructs

All constructs were synthesized as gene fragments using sequences available in the GenBank database. The YF17D backbone constructs were synthesized based on accession number X03700 (previoulsy K02479). Eight Yellow fever 17D-DENV chimeric constructs were designed, the details of which are presented in Table 1. Four of these constructs were designed to be similar to the four CYD vectors in Dengvaxia® [18] in order to serve as comparators for our tetravalent DEN-80E subunit vaccine (V180) [21]. The remaining four constructs were designed based on extensive analysis of dengue sequences available in GenBank. Full-length prM and E sequences of each dengue serotype (866 sequences for DEN1, 735 sequences for DEN2, 358 sequences for DEN3 and 75 sequences for DEN4) were analyzed to derive a consensus prM and E sequence for each serotype. prM and E sequences from naturally occurring isolates closest to each of the consensus sequences were selected for vector construction. Only sequences from isolates reported after 1987 were selected with the rationale of using more recent strains as vaccine components. We also attempted to select geographically-dispersed dengue virus strains.

**Table 1:** Recombinant chimeric Yellow fever dengue constructs.

Virus construct	Reference†	Place of isolation	Year isolated	Difference from consensus E (aa changes)
rYFD1-PUO359	[17]	Thailand	1980	10
rYFD1-V4033	ACY70690	Vietnam	2008	0
rYFD2-PUO218	[17]	Thailand	1980	7
rYFD2-V1168	ACA48839	Puerto Rico	1987	1
rYFD3-PaH881/88	[17]	Thailand	1988	10
rYFD3-V3929	ACW82935	St. Lucia	2001	0
rYFD4-1228 (TVP-980)	[17]	Indonesia	1978	6
rYFD4-MY01-23476	CAZ72181^‡^	Malaysia	2001	5

†—Accession number is provided in the absence of a publication

‡—Record has since been removed from GenBank

### Generation of YF-17D dengue infectious clones and LONG-PCR

**Fig 1:**
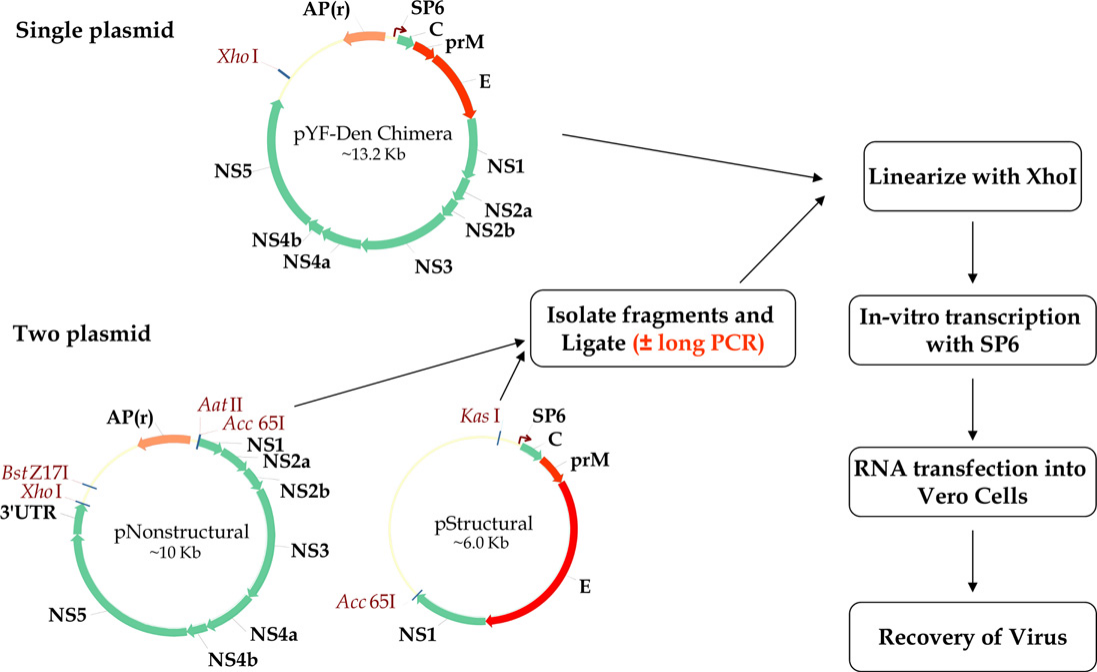
Outline of recombinant YF-17D-dengue chimeric virus recovery. The chimeric constructs were designed such that recombinant viruses can be recovered either by the single-plasmid (linearized single plasmid) or the two plasmid approach (*in-vitro* ligation of DNA fragments). The full-length chimeric cDNA clone or *in-vitro*-ligated chimeric DNA was linearized with XhoI to allow for run-off transcription. A LONG-PCR step was also performed in some cases to generate templates for *in vitro* transcription. Chimeric full-length viral RNA transcripts thus generated were transfected into Vero cells for virus recovery.

Viral genomes flanked upstream by the SP6 promoter were synthesized as gene fragments and sequentially cloned into low-copy number plasmid pACYC177 (NEB). However, in spite of evaluating several bacterial hosts (DH10B, XL-1 blue, Sure2, Stbl2 and Stbl3), full-length infectious cDNA clones could only be generated for 3 of the 8 planned constructs due to plasmid stability issues. For the remaining 5 constructs, a two-plasmid approach which involved in vitro ligation of two cDNA fragments was attempted (Fig 1). Briefly, plasmids (pStructural) containing the 5’ UTR and capsid region of YF17D and, the prM and E genes of each DENV serotype were generated. A second plasmid (pNonstructural), containing the remaining non-structural genes and 3’ UTR of YF 17D was also produced. Plasmids pStructural and pNonstructural were digested separately using KasI-Acc65I and Acc65I-BstZ17I enzyme combinations, respectively, to yield the appropriate fragments. These fragments were then agarose gel-purified using a QIAquick gel extraction kit (QIAGEN), ligated using T4 DNA ligase (NEB) and then used as templates for in vitro transcription reactions (IVT) using an mMessage mMachine SP6 transcription kit (Life Technologies). If constructs could not be recovered into recombinant viruses using the above approach, ligated DNA templates were PCR amplified using Phusion Hot Start II DNA polymerase (NEB) in order to amplify the full-length genome. Reaction parameters for LONG-PCR were as follows: 98^0^ C for initial denaturation (30 sec), 25 cycles of 98^0^ C (denaturation, 10 sec) and 72^0^ C (annealing and elongation, 5.5 min). The final extension time was 10 min at 72^0^ C. The amplified PCR product then served as template for the IVT reaction. All LONG-PCR products were sequenced in their entirety before being used in *in vitro* transcription reactions, to confirm that no sequence changes were introduced during the PCR step.

### Recovery of recombinant CYD viruses

Linearized plasmid DNA from full-length clones or LONG-PCR products generated from two plasmid ligation reactions were used as template for RNA transcription. Briefly, the extracted plasmid DNA (single plasmid) or LONG-PCR product (generated from 2-plasmid ligation as template) was digested with XhoI and used in a SP6-RNA polymerase catalyzed IVT reaction (40ul volume). An aliquot (15ul) of the RNA transcript was run on a glyoxal RNA gel to check the length and the integrity of the synthesized RNA. An equal volume of the RNA transcript was transfected into Vero cells for recombinant virus recovery. Transfections were carried out using 30ul of Lipofectamine 2000 (Invitrogen), according to the manufacturer's directions. The transfection mixture was removed after overnight incubation at 37°C, and the cells were washed and maintained in 3 ml of medium 199 containing 2% fetal bovine serum. In some cases, a duplicate transfection well was overlaid with 1% methyl cellulose after the wash to assess RNA infectivity. Transfected cells were monitored daily for virus-induced cytopathic effect (CPE). After a 5-day incubation, the transfected cells were scraped into the medium (Passage 0) and the entire harvest was passed onto Vero cells grown in T-25 flasks for further amplification (Passage 1). Four to six days post-transfer, the supernatant was harvested and the recovery of recombinant virus was confirmed by RT-PCR and immuno-fluorescence. For a master stock, one additional passage (Passage 2) was performed and the supernatant was harvested and clarified for further use. The recovered recombinant virus stocks were also completely sequenced at passage 1 to confirm their identity and verify the complete sequence.

### Plaque assay

Plaque assays to determine virus titers were performed on 6-well plates containing confluent Vero cells. Cell monolayers were incubated with 10-fold serial virus dilutions for 1 h at 37°C. After virus adsorption, the inoculum was removed and replaced with 3 ml of Medium 199 containing 2% fetal bovine serum and 1.0% methyl cellulose (Sigma), and the cells were incubated at 37°C. After a 5-day incubation, the methyl cellulose overlays were removed and the cells were fixed and stained with 80% methanol/crystal violet solution for 2 h at room temperature. Following fixing and staining, the plates were washed and allowed to dry. Viral plaques were then counted to determine virus titers and reported as plaque forming units (PFU) per ml.

### Immunofluorescence

Verification of virus recovery was performed using immunofluorescence on Vero cells grown in chamber slides. A small (~ 100ul) of the transfection supernatant was transferred onto the Vero cells. After incubation for 4 days at 37°C, the media in the chamber was removed and the monolayer was washed with PBS and then fixed with 80% acetone. The fixed cells were then incubated at 37^0^ C with 1:1000 dilution of a pan-flavivirus monoclonal antibody, 4G2 [22] for 1 h. The cells were then washed and incubated with 1:1000 dilution of fluorescein isothiocyanate (FITC)-labeled goat anti-mouse IgG (Life Technologies) for 45 min. Finally, the monolayers were washed and covered with a coverslip and observed under a fluorescent microscope (Zeiss).

### Rhesus monkey studies

Healthy adult, Indian rhesus macaques of either sex, weighing more than 3 kg, and which were flavivirus (DENV 1, 2, 3 and 4, and WNV) antibody-negative by virus neutralization titer (for DENV) and ELISA (for WNV) were utilized in this study. The animals were sedated with Ketamine (5–30 mg/kg, IM) for vaccination, and blood collection. Two groups of 4 monkeys each were used in this study and all animals were immunized at 0 and 6 months. Animals in group 1 were immunized with saline via the intramuscular route while those in group 2 were immunized with a tetravalent formulation of selected CYD containing 10^5.0^ PFU of each individual chimeric virus via the subcutaneous route. After vaccination, the animals were observed daily for any changes at the inoculation site or other changes in activity or feeding habits that might indicate an adverse reaction to the vaccine. Blood samples from the immunized animals were collected at monthly intervals over a period of 9 months and, used in an Infra-red dye (IRD)-based LiCor dengue neutralization assay to evaluate immunogenicity.

### LiCor dengue neutralization assay

The LiCor dengue neutralization assay is a sensitive, high-throughput Infra red dye (IRD)- based neutralization assay that was developed and utilized to determine all dengue neutralization titers [21]. Briefly, two-fold serial dilutions of heat-inactivated serum samples from vaccinated animals were incubated for 1 h at 37°C with 50 PFU of each DENV. This virus-serum mixture was then added onto Vero cells in 96-well plates and incubated for 4 days. Following incubation, the culture media was removed, the cells were fixed with 3.7% formaldehyde in PBS and permeabilized using 0.1% Triton X-100/PBS. Immunostaining of the plates was carried out using the pan-flavivirus 4G2 antibody as the primary antibody and a biotinylated horse anti-mouse IgG (Vector Laboratories, CA) as the secondary antibody. The secondary antibody was followed by the addition of a cocktail of IRDye^®^ 800CW Streptavidin (1:1000, LiCor Biosciences) and DRAQ5 (1:10,000, Shepshed, Leicestershire, UK). The DRAQ5 stain was used to stain Vero cell DNA for normalization in the IRD assay. After the final incubation, the mixture was removed and the plates were washed, air-dried and scanned using an infrared Odyssey^®^ Sa imaging system (Li-Cor Biosciences). Serum end-point neutralization titers (averaged from duplicate wells) were defined as the reciprocal of the highest serum dilution that reduces the 800nm/700nm fluorescence integrated intensity ratio greater than 50% when compared to virus control included on each assay plate. In order to calculate group geometric mean, a titer of 1:5 was assigned to samples without any neutralization titer.

## Results and Discussion

### Chimeric Yellow fever-dengue constructs

In this study, a total of 8 YF17D-DENV chimeric constructs were designed (Table 1). Four of these constructs were designed to be identical to the Dengvaxia® vaccine developed by Sanofi Pasteur [18] in order to serve as comparators for our candidate tetravalent subunit dengue envelope vaccine (V180) [21]. The remaining four constructs were designed based on an extensive analysis of a total of 2034 full-length prM and E sequences available in GenBank for all four dengue serotypes. Consensus prM and E sequences for each serotype were derived and a naturally-occurring sequence, closest to each of the consensus prM and E, was selected for our constructs. The analysis focused on more recent (viruses isolated post 1987) and geographically-dispersed dengue virus strains. The prM and E sequences of DEN1, DEN2, DEN3 and DEN4 viruses in Dengvaxia® were all from viruses isolated in southeast Asia i.e. Thailand and Indonesia. In contrast, our constructs contained prM and E sequences of DEN1, DEN2, DEN3 and DEN4 viruses isolated from Vietnam, Puerto Rico, St. Lucia and Malaysia respectively, representing a much broader geographic distribution. Furthermore, the viruses in Dengvaxia® were mostly isolated in the 1980s while 3 of our 4 (DEN1, DEN3 and DEN4) viruses were isolated in the 2000s, representing more recent virus strains. Our DEN2 component was based on a virus isolated in 1987 in Malaysia (Table 1). Finally, the prM and E sequences in Dengvaxia® differed from our derived consensus sequences by 6–10 amino acids (aa) while the sequences in our constructs differed by only 0–5 aa (Table 1). We believe that inclusion of recently-isolated and geographically-dispersed sequences as vaccine components could offer better coverage against circulating viruses. However, whether these “closer-to-consensus” sequences offer any advantage in terms of broader immunogenicity or protection remains to be studied. All the virus constructs were synthesized as gene fragments for sequential assembly into full-length clones or subclones.

### Generation of full-length cDNA clones

Initially, we planned to generate a full-length YF17D cDNA clone that could be used to generate the CYD plasmids. However, as experienced by other groups, a full-length infectious cDNA clone of YF17D could not be successfully generated in spite of evaluating several bacterial competent cells such as DH10B, XL-1 blue, Sure2, Stbl2 and Stbl3. As the CYD infectious clones were our ultimate objective, we resorted to generating the chimeric plasmids directly. The CYD constructs were synthesized as four individual cDNA fragments: fragment 1 contained the 5’ UTR and capsid genes of YF17D, the prM and E genes of the respective DENV, and partial sequence of the NS1 gene of YF17D. This fragment was flanked by a SP6 RNA polymerase promoter upstream and an Acc65I site at the downstream end. Fragment 2 contained the remainder of NS1, NS2a, NS2b and part of the NS3 genes of YF17D and was flanked by an Acc65I site upstream and a BssHII site downstream. Fragment 3 contained the remaining portion of NS3, NS4a, NS4b and partial NS5 genes of YF17D and contained a BssHII site upstream and a BspEI site downstream. Fragment 4 contained the remaining sequences of the NS5 gene and the 3’ UTR sequence of YF17D, flanked by the BspEI site upstream and an XhoI site downstream. The Acc65I site was a unique site naturally occurring in the NS1 gene of the YF17D genome, while the BssHII and BspEI sites were introduced via 3- and 2-nt substitutions, respectively, to assist in cloning and also serve as genetic markers in the recombinant viruses. A unique XhoI site was included at the end of the genome for use in linearizing the plasmid prior to in vitro transcription. Additionally, the cleavage site of XhoI (C↓TCGAG) corresponds to the terminal “C” nucleotide of the viral genome.

**Fig 2:**
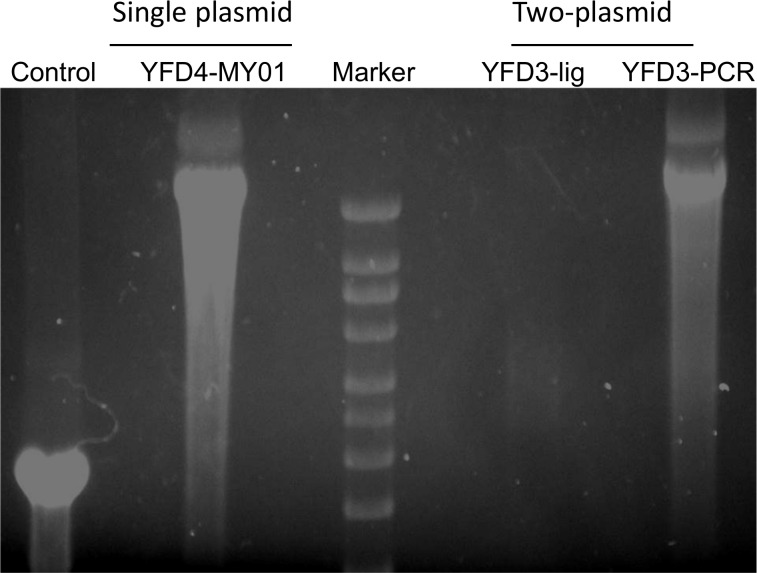
RNA transcripts from LONG-PCR template. *In vitro* transcription reactions were performed using DNA templates from single-plasmid or two-plasmid (ligation/non-PCR and ligation/LONG-PCR) constructs. Equal volume aliquots of RNA transcripts produced were denatured at 50^0^ C for 30 minutes and then were electrophoresed on a 1% denaturing glyoxal gel. Control lane indicates RNA transcript produced from the control DNA template included in the in vitro transcription kit.

Among the 8 CYD constructs designed, full-length clones of three chimeras (YFD2-PUO218, YFD4-1228 (TVP-980) and YFD4-MY01-23476) were successfully generated by sequential cloning of the respective four individual cDNA fragments into the low-copy number plasmid (pACYC177) using SURE2 competent cells (Table 2). These full-length plasmids were sequenced in their entirety and were faithful copies of the CYD constructs, except for the artificially-introduced genetic markers. The remaining 5 CYD constructs could not be generated as full-length clones in spite of trying several different bacterial cells and transformation conditions. For these constructs, it was decided to follow the two-plasmid system as shown in Fig 1. For this, fragment 1 was cloned into a pUC57-based plasmid and was denoted as pStructural while, fragments 2, 3 and 4 were cloned together into pACYC177 in a sequential manner to yield the pNonstructural plasmid. Interestingly, fragments 2, 3, and 4 were stable in the pNonstructural plasmid (Fig 1). The pStructural plasmid was digested using KasI and Acc65I enzymes and the desired fragment was gel-purified. Similarly, the desired fragment resulting from Acc65I-BstZ17I digestion of the pNonstructural plasmid was also gel-purified. However, when equal concentrations of the two fragments were used in a ligation reaction, the ligation product yield was inconsistent and often contained multiple ligation products of different sizes. This mixed population of ligation templates yielded little to no RNA of the desired size when used as template in in-vitro transcription (IVT) reactions (Fig 2). Only in the case of the YFD1construct, following several attempts of ligation-IVT, were we able to observe a visible band of the expected size on a RNA gel, and using this product, we were able to successfully recover infectious virus.

**Table 2:** Summary of recombinant viruses recovered.

Recombiant Virus	Rescue method^†^	Sequence change in recombinant virus	Sequence change in LONG-PCR product
rYFD1-PUO359	Two-plasmid	5651 (NS3 gene) CTA to TTA (silent); 7319 (NS4b); GAG to AAG (Glu-Lys)	N/A
rYFD1-V4033	Two-plasmid (Long-PCR)	7319 (NS4b); GAG to AAG (Glu-Lys)	None
rYFD2-PUO218	Single-plasmid (SURE2 cells)	7319 (NS4b); GAG to AAG (Glu-Lys)	N/A
rYFD2-V1168	Two-plasmid (Long-PCR)	7319 (NS4b); GAG to AAG (Glu-Lys)	None
rYFD3-PaH881/88	Two-plasmid (Long-PCR)	7319 (NS4b); GAG to AAG (Glu-Lys)	None
rYFD3-V3929	Two-plasmid (Long-PCR)	7319 (NS4b); GAG to AAG (Glu-Lys)	None
rYFD4-1228 (TVP-980)	Single-plasmid (SURE2 cells)	7319 (NS4b); GAG to AAG (Glu-Lys)	N/A
rYFD4-MY01-23476	Single-plasmid (SURE2 cells)	None	N/A

† *E*. *coli* competent cells are provided in the case of successful single plasmid rescue method

Due to the inconsistency of the ligation reactions and resulting failure to rescue recombinant viruses, we decided to perform LONG-PCR to amplify and enrich the template for the IVT reactions. For the final 4 constructs, ligation products were subjected to LONG-PCR using Phusion Hot Start II DNA polymerase (NEB) which consistently yielded a specific amplicon of the expected size. It is interesting to note that when sequenced, the LONG-PCR amplicons of all four constructs contained the expected sequence without any artificially-introduced mutations indicating high fidelity of the DNA polymerase used in the LONG-PCR. These amplicons were then used in IVT reactions and the resulting RNA transcripts were used for virus recovery. The LONG-PCR-amplified templates yielded the right size RNA transcripts almost to the same levels as those derived from single plasmid templates (Fig 2), indicating the advantage offered by the PCR amplification of the ligation product.

### Recovery of infectious recombinant viruses

**Fig 3:**
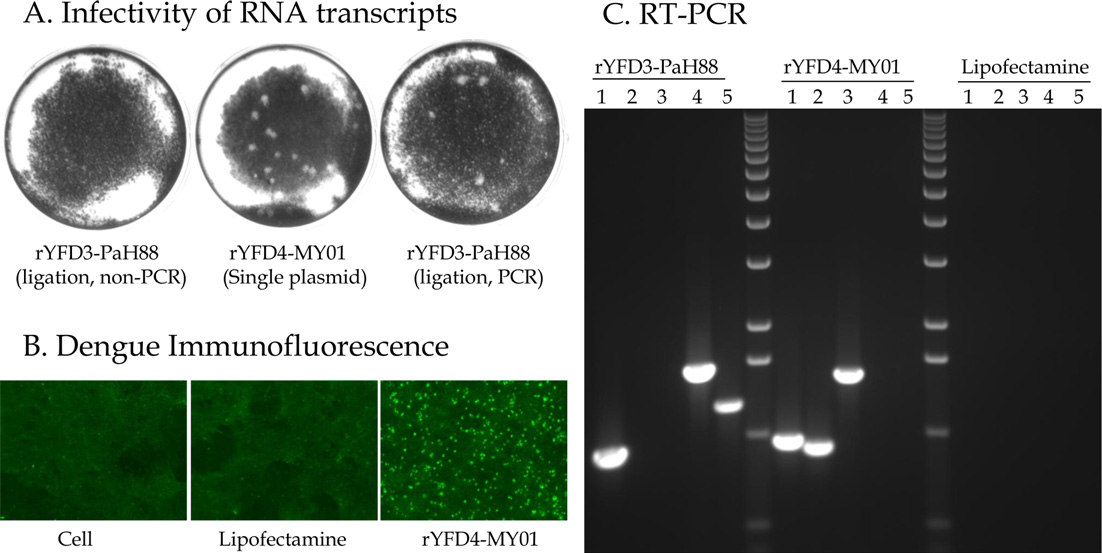
Confirmation of recombinant virus recovery. **A**. Infectivity of RNA transcripts was determined in Vero cells. **B**. Virus recovery was also confirmed by immunostaining using pan-flavivirus antibody 4G2. **C**. Virus recovery and identity was confirmed by RT-PCR using primers specific to YF (1), D4-MY01 (2), D4-MY01/YF junction (3), 5’ YF/D3 junction (4) and 3’ YF/D3 junction (5).

The RNA transcripts from all 8 YFD constructs were transfected individually into Vero cells. Parallel transfections were performed and overlaid with 1% methyl cellulose to determine RNA infectivity. Transfected cells were observed daily and they started showing dengue virus-like CPE around 4–6 days post transfection. At around the same time point, plaques similar to those produced by dengue virus could be observed in RNA transfections with methyl cellulose overlay (Fig 3A). These observations collectively indicated recovery of infectious recombinant CYD viruses from the transfections. In general, when cells were transfected with IVT RNA derived from a single plasmid template, recombinant virus was readily recovered. This was the case for the YFD2-PUO218, YFD4-1228 (TVP-980) and YFD4-MY01-23476 constructs (Table 2). In the case of the YFD1-PUO359 construct, virus recovery was achieved using the two-plasmid ligation method (without LONG-PCR) after at least 5 separate attempts. Additionally, this method required several empirical modifications to ligate the two fragments and to generate RNA transcript of the required quality through IVT. The additional work required for the successful recovery of this virus emphasizes the technical difficulty of the two-plasmid system. In the case of the YFD-PaH881/88 construct, the two-plasmid ligation method (without LONG-PCR), did not yield any virus in spite of several (n = 7) attempts. These challenges underscore the importance of a full-length DNA template for efficient RNA transcript production and virus recovery. Therefore, we used the LONG-PCR method for the remaining 4 constructs i.e. YFD1-V4033, YFD2-V1168, YFD3-PaH881/88 and YFD3-V3929 (Table 2). Using the LONG-PCR approach, we were able to successfully recover all 4 constructs on the first attempt, demonstrating the advantage of including the PCR step to amplify the template prior to IVT.

The supernatants from each of the transfections upon further passage onto fresh Vero cells showed DENV-like CPE and were also positive for dengue virus specific immunostaining when tested with a pan-flavivirus antibody 4G2 (Fig 3B). Furthermore, the identity of the recovered viruses was confirmed by RT-PCR using primers specific to the YF17D backbone, DENV backbone and different YF-dengue junctions for each serotype (Fig 3C). Direct DNA sequencing of the RT-PCR products was also carried out. Collectively, these findings confirm that the viruses recovered through these methods are authentic recombinant CYD viruses. All the recovered CYD viruses were grown in Vero cells for two additional passages. The peak titer for all viruses was between 10^5^ and 10^6^ PFU/ml when harvested between 5–6 days post-infection.

The complete genome consensus sequence of each of the 8 CYD viruses (P1) was determined from uncloned RT-PCR fragments generated from viral RNA extracted from Vero cells. Table 2 provides the summary of sequence changes observed in the recovered recombinant CYD viruses. None of the prME regions in any of the CYD viruses contained sequence changes indicating that the dengue sequences in these chimeric viruses are intact, even in the context of a chimeric backbone. On the other hand, analysis of sequences of the YF virus genes revealed that 7 out of 8 CYDs contained 1–2 nucleotide (nt) changes (Table 2). Strikingly, one mutation (GAG to AAG) was observed in the NS4b gene of all 7 constructs, resulting in an amino acid change from glutamine to lysine (E to K) at position 7319. This mutation did not appear to be associated with the method of virus recovery. Interestingly, this mutation was not present in the plasmid DNA or LONG-PCR DNA and therefore, is probably the result of virus adaptation to Vero cells. Furthermore, this same mutation has been observed in several other YF17D-based chimeric flaviviruses [23, 24], indicating that it is probably a virus-associated change and not an artificially-induced one. In the case of rYFD1-PUO359 virus, an additional mutation (CTA to TTA) was observed in the NS3 gene at position 5651, which did not result in any amino acid substitution. Only the rYFD4-MY01-23476 virus, rescued using the single plasmid method, completely matched the cDNA sequence. Together, these results suggest that the LONG-PCR method in addition to offering a significant advantage to virus recovery does not result in the introduction of additional mutations into the viral genomes of the viruses generated.

### Immunogenicity of recovered recombinant viruses

It is believed that a dengue vaccine should be tetravalent in nature so that it will provide robust protective immunity against all four serotypes simultaneously and not sensitize people for DHF/DSS [25, 26]. Although there is no validated disease model for dengue, the rhesus macaque model is well-recognized as the most relevant model for assessing dengue vaccine immunogenicity and efficacy. Therefore, we decided to evaluate the immunogenicity of the recovered CYDs as a tetravalent formulation in rhesus macaques. A group of 4 monkeys served as a negative control group and were administered saline. Another group of 4 monkeys were immunized with a tetravalent formulation of 10^5.0^ PFU of each selected CYD (rYFD1-PUO359, rYFD2-PUO218, rYFD3-PaH881/88 and rYFD4-1228(TVP-980)) via the subcutaneous route (0.5ml/dose). Immunizations were performed at 0 and 6 months to be consistent with the vaccination regimen followed for the live attenuated Dengvaxia® vaccine. Serum samples were collected at monthly intervals post vaccination and assessed for levels of neutralizing antibodies against each of the four dengue serotypes using a LiCor assay [21].

**Fig 4:**
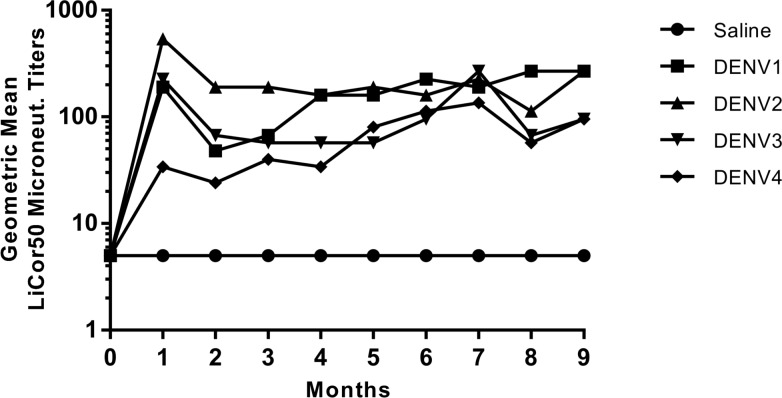
Immunogenicity of CYD viruses in rhesus monkeys. Longitudinal geometric mean virus neutralization titers in rhesus monkeys immunized with a tetravalent formulation of recombinant CYD viruses. A group of 4 monkeys was immunized with a tetravalent formulation containing 10^5^ PFU of each of rYFD1-PUO359, rYFD2-PUO218, rYFD3-PaH881/88 and rYFD4-1228 (TVP-980) viruses via the subcutaneous route. A negative control group of 4 monkeys was included that received saline via intramuscular route. Both groups were immunized at 0, and 6 months. Virus neutralizing antibody titers were measured over a period of 9 months using a LiCor-based neutralization assay. The geometric mean virus neutralization titers at each time point are presented representing the virus neutralization results for DENV1, DENV2, DENV3, and DENV4.

In the case of animals immunized with the tetravalent CYDs, neutralizing antibodies to each of the dengue viruses could be detected one month post the first dose, as would be expected from a LAV vaccine (Fig 4). The geometric mean titers (GMT) at 1 month post dose 1 were 190, 538, 226 and 34 against DENV1, DENV2, DENV3 and DENV4 respectively. These titers declined slightly at study month 2 but then remained steady until the 6 month boost. A second dose of the same tetravalent formulation at 6 months resulted in a slight boost in DENV3 titers but not for any of the other serotype titers, indicating sterilizing immunity resulting from the first dose. The GMT at study month 7 (1 month post-boost) was 381, 226, 269 and 135 against DENV1, DENV2, DENV3 and DENV4 respectively. Furthermore, the tetravalent CYD formulation was also observed to induce a balanced and durable immune response against each serotype for the entire duration of the 9-month study period (Fig 4, S1 Table). These results indicate that the recovered recombinant CYDs are immunogenic and can induce a well-balanced, durable immune response against all four serotypes when given as a tetravalent combination without exhibiting any viral interference.

## Conclusion

The use of reverse genetic systems has facilitated the basic discovery of and vaccine development for a number of RNA viruses. Full-length infectious clones are currently available for several flaviviruses [27]. A single full-length cDNA clone is preferred for efficient flavivirus engineering and recovery since it offers a consistent template for generating high quality viral RNA transcripts resulting in a significantly higher success rate for virus recovery. However, genetic instability of full-length cDNA in *E*. *coli* has proved to be a significant hurdle to molecular studies and vaccine development for some flaviviruses. Infectious clones of YF17D and JEV strain JaOArS982 were prepared using a two-plasmid system that involves ligation of the 5’ and 3’ halves of the respective genomes prior to transcription to generate RNA for virus rescue [9, 28]. There is even an example of having to use a three-fragment ligation strategy to overcome stability issues in *E*. *coli* during the generation of an YF17D/DENV3 chimera [15]. The instability of full length cDNA clones in *E*. *coli* has spurred the development of alternative strategies such as a yeast-*E*.*coli* shuttle vector and homologous recombination to assemble a DENV3 genome, which was then propagated in yeast [12]. Two-plasmid or multi-plasmid systems offer both advantages and disadvantages. Multi-plasmid systems can be faster and more versatile to use since full-length clone generation is avoided and the use of subclones/fragments can make genome manipulation or mutagenesis easier. Using appropriate design, this methodology can be applied to the generation of multiple chimeric viruses with a common 3’ or 5’ half. But the major drawback of the two/three-plasmid systems that we encountered is the poor quality and quantity of RNA transcripts that are generated using the template derived from ligation of the component fragments.

In an attempt to improve this step in virus recovery we have developed a novel LONG-PCR strategy to amplify and enrich the full-length templates of YF17D-dengue chimeric genomes prior to transcription. Our method combines the advantages of both the single and two-plasmid systems and hence resulted in rapid generation of recombinant flaviviruses with high efficiency. We recovered 4 of our 8 recombinant CYDs using this method on the first attempt without introducing any sequence changes in the recovered recombinant viruses. These chimeras behaved like authentic viruses and were immunogenic in rhesus macaques, indicating that vaccine virus candidates can be generated rapidly using the LONG-PCR method. Furthermore, with the availability of DNA polymerases with very high proof-reading abilities, the LONG-PCR step can be included to generate templates without concerns about unwanted sequence changes. Long range RT-PCR has been described previously for different enteroviruses [29–31]. This work is the first to demonstrate that infectious recombinant flaviviruses can be rescued through long range PCR in a rapid manner. In conclusion, we believe that the modified virus rescue strategy using LONG-PCR is a quick and simple method to efficiently generate flaviviruses for use in basic research studies or for development as vaccine candidates.

## Supporting Information

S1 Table Neutralization titers in immunized monkeys. (XLSX)
